# Salivary Metabolome and Soccer Match: Challenges for Understanding Exercise induced Changes

**DOI:** 10.3390/metabo9070141

**Published:** 2019-07-11

**Authors:** Erica Pitti, Greta Petrella, Sara Di Marino, Vincenzo Summa, Marco Perrone, Stefano D’Ottavio, Andrea Bernardini, Daniel Oscar Cicero

**Affiliations:** 1 Department of Chemical Science and Technologies, University of Rome Tor Vergata, 00133 Rome, Italy; 2 IRBM S.p.A., 00071 Pomezia, Italy; 3 Department of Experimental Medicine and Surgery, University of Rome Tor Vergata, 00133 Rome, Italy; 4 Department of Clinical Sciences and Translational Medicine, University of Rome Tor Vergata, 00133 Rome, Italy

**Keywords:** metabolomics, sport metabolomics, NMR, exercise, saliva, energy metabolism, hydration status

## Abstract

Saliva samples of seventeen soccer players were analyzed by nuclear magnetic resonance before and after an official match. Two different ways of normalizing data are discussed, using total proteins and total metabolite concentrations. Changes in markers related to energy, hydration status, amino acids and other compounds were found. The limits and advantages of using saliva to define the systemic responses to exercise are examined, both in terms of data normalization and interpretation, and the time that the effect lasts in this biofluid, which is shorter to that commonly observed in blood. The heterogeneous nature and different timing of the exercise developed by players also plays an important role in the metabolic changes that can be measured. Our work focuses mainly on three different aspects: The effect that time sampling has on the observed effect, the type of normalization that is necessary to perform in order to cope with changes in water content, and the metabolic response that can be observed using saliva.

## 1. Introduction

After recent advances in big data management, considerable effort was devoted to the search of biomarkers in the field of sports and recreational exercise [1]. Nowadays, there are an increasing number of commercially available services offering biochemical and genetic testing for players. Professional athletes are starting to exploit technology and biomarker testing to follow performance and recovery during training. Assessing health, sport, performance and recovery of athletes, which represent a broad physiological function, requires monitoring simultaneously a large number of biomarkers. This makes the use of metabolomics particularly interesting, as opposed to the measurement of a small number of metabolites, normally considered as unique biomarkers.

Regarding the specific field of soccer activity, some recent studies have observed the metabolic changes that result from participation in a soccer match [2,3,4,5,6,7,8]. Considerable attention has also been placed on the use of saliva as a potentially useful biofluid to follow metabolic changes during exercise [9]. Saliva offers a number of advantages with respect to the more traditional blood sampling. Salivary biomarkers are collected in a non-invasive fashion, causing only minimal distress to the individuals. Its collection requires neither laboratory facilities nor skilled healthcare professionals [10]. On the other hand, saliva shows two main disadvantages with respect to blood: It reflects both metabolites from the body as well as those from bacteria living in the oral cavity, like butyrate and the enantiomeric forms of amino acids; and substances arising from food, and the time that an effect lasts after exercise can be shorter. For example, salivary pH, salivary secretion rates, protein concentration and protein secretion rates, have all returned to their basal values after 30 min of recovery [11]. Salivary lactate returned to basal levels eight minutes after exercise was stopped, whereas blood lactate was still 3.5 times higher after 20 min [12]. This is particularly relevant when samples are analysed after a soccer match, because different players can rest differently after the last sprint or run, making the variation in salivary biomarkers very heterogeneous among the group or hardly detectable for certain individuals.

Understanding changes in metabolite concentrations in saliva occurring during a soccer match meets a significant additional challenge, that is, the nature of the exercise, which is far from controlled and homogeneous for all players. Most of the literature regarding correlation between metabolite levels and physical effort were obtained under controlled and homogeneous conditions for all subjects involved in the studies. On the contrary, soccer is known to be an intermittent sport, in which players exert a physical activity at various intensities distributed in an a-cyclical mode. In this work, saliva samples have been tested using nuclear magnetic resonance (NMR). In this way, changes in the concentration of 56 metabolites after an official soccer match could be followed simultaneously on saliva samples of 17 female professional players. Only a few works dealing with salivary biomarkers and soccer refer to female players [7,8], so specific markers taking into account biological sex can add significant information. Our work focuses mainly on three different aspects: The effect that time sampling has on the observed effect; the type of normalization that is necessary to perform in order to cope with changes in water content; and the metabolic response that can be observed using saliva. These results can be useful to explore the limits and advantages of the use of saliva to detect systemic responses that are promoted by this specific form of physical activity.

## 2. Results

### 2.1. Saliva Biomarkers Quantified by NMR

The main characteristics of the 17 female professional team soccer players participating in the study and involved in an official match are reported in Table 1. To perform the analysis, players were divided into four groups: Those participating in the entire game (game group, n = 8); those that were substituted (exit group, n = 3, average on-field time = 71 ± 17 min); those that entered (enter group, n = 3, average on-field time = 19 ± 16 min) and finally those that did not participate (no game group, n = 3).

**Figure 1:**
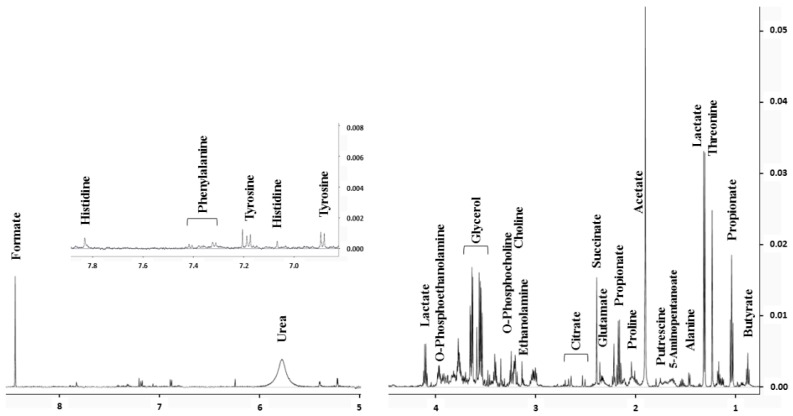
Nuclear magnetic resonance (NMR) spectrum of a saliva sample with the assignment of major metabolites.

By using NMR to quantify saliva biomarkers, it is possible to measure the concentration of 56 metabolites from a single spectrum of filtered saliva (see material and methods). Figure 1 shows a typical spectrum with the assignment of major metabolites and Table 2 shows the list of metabolites quantifiable using this technique.

Additionally, this study also measured total protein concentration of whole saliva (TPWS) and estimated the total observed metabolite concentration (TOMC), which is correlated to saliva osmolality, a relevant marker of hydration status.

### 2.2. Total Proteins (TPWS), Total Detected Metabolite Concentration (TOMC) and Urea Nitrogen/Creatinine Ratio as Indexes of Exercise Load and Hydration Status

**Figure 2:**
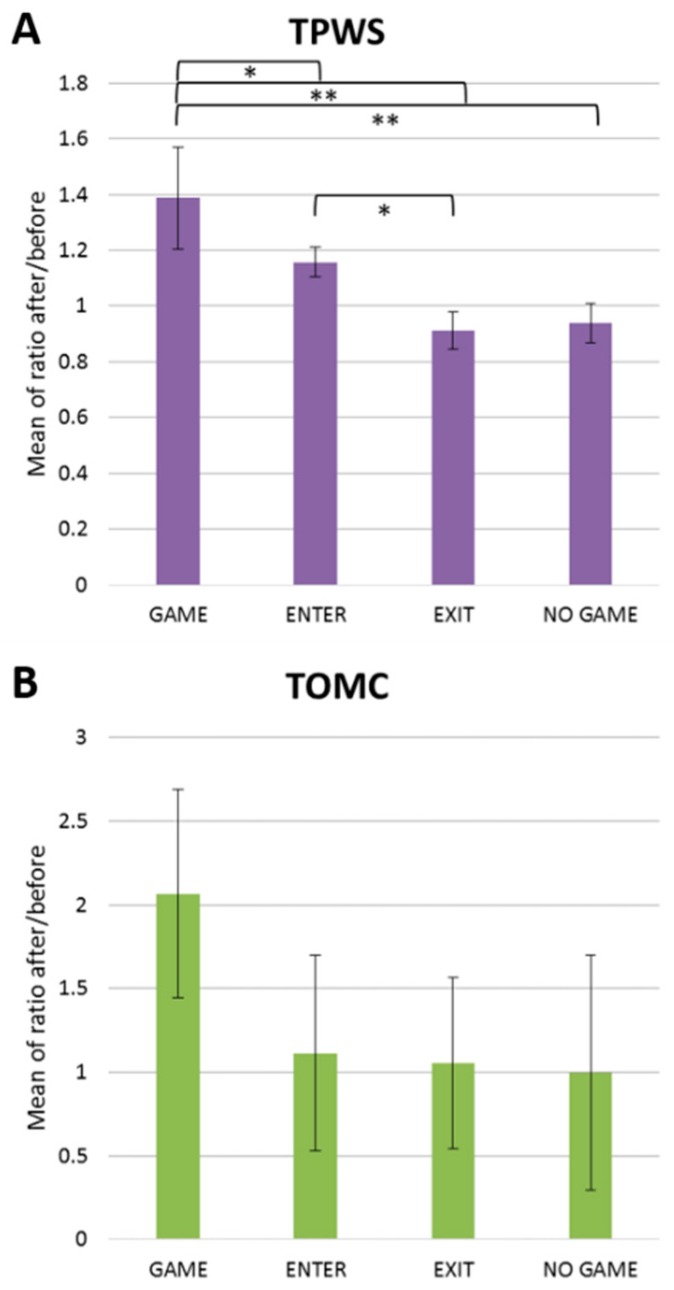
The average values of after/before match ratios referring to (**A**) total proteins (TPWS) and (**B**) total observed metabolite concentrations (TOMC).

Different biomarkers are commonly used to follow exercise load and hydration status. Among them, TPWS was shown to present a moderate increase after aerobic exercise and a strong increase after intense exercise [11]. The authors measured TPWS pre-/post-game ratio for the four groups and results are shown in Figure 2A. A significant increase for the game group was noticed, in the order of 40%, and a moderate increase for the enter group of approximately 20%. No increase was observed for the exit or no-game group. This result showed, on one hand, that TPWS correlated well with the total game time and, on the other hand, players that were substituted returned to basal levels before the post-game sample was collected.

The observed increment of TPWS in the game and enter groups can be caused by a combined action of water content loss and stimulation of the sympathetic nervous system [13]. However, linked to these effects is the TOMC, whose values are correlated to salivary osmolality that, in turn is recognized as an efficient biomarker for hydration status [14]. Figure 2B shows the TOMC pre-/post-game ratio for the four groups. There is a clear trend showing an increase in TOMC between 80 and 100% in the game group. However, the mean values were not statistically significant with respect to the other three groups that showed average ratios of approximately 1.0, mainly due to a large variation among the individuals. As in the TPWS normalization, both the exit and no-game groups showed no significant differences in the total metabolite levels between pre- and post-samples. Based on these results, the authors defined a group called normally hydrated (NH), which comprised both the exit and no-game groups. For these individuals, it was expected that there were minimum changes in water content, either because they did not play, or because the resting period was too long and the dehydration effect vanished. In this way, this study has a comparable number of players in the NH group (n = 6) and in the game group (n = 7) to draw some conclusions about the change in hydration status.

**Figure 3:**
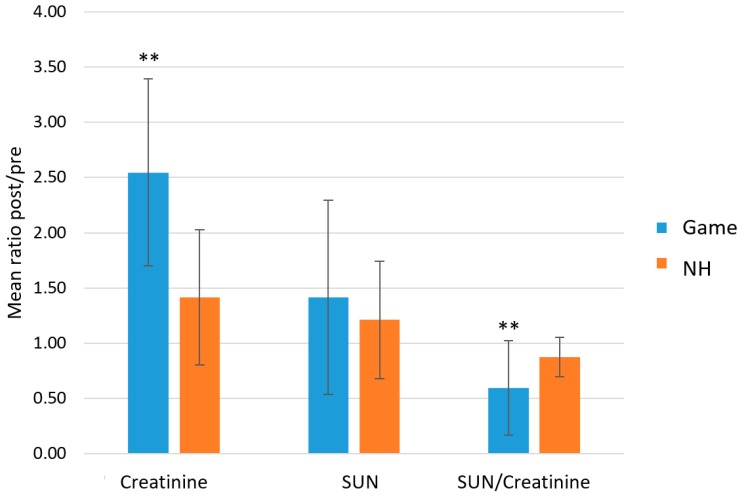
The ratio between post- and pre-game samples for creatinine, urea nitrogen (SUN), and the ratio SUN/creatinine for the game group and the normally hydrated (NH) group. The last includes both no-game and exit groups. ** *p* < 0.01. p values were obtained between pre- and post-samples of both game and NH groups. The last comprises both the exit and no-game groups (see text).

An index normally used to assess dehydration is the blood urea nitrogen/creatinine ratio [14]. A value above 20 is considered an index of dehydration or hypoperfusion. The authors have investigated if this ratio in saliva is also sensitive to the hydration status. Table 3 shows the normal values for serum and saliva, both from this work and other works present in the literature [15]. The saliva urea nitrogen (SUN)/creatinine ratio was lower than the lower limit accepted for the corresponding blood index. The game-induced variations in SUN was then measured, along with creatinine and the ratio SUN/creatinine in the game group and the previously defined NH group formed by the exit- and no-game groups, which showed little variation in TPWS and TOMC (see above). The results are shown in Figure 3. The game group showed a non-statistically significant increase in SUN and a significant increase in creatinine in the post-game samples. As a result, there was a significant decrease in the SUN/creatinine index after exercise. The NH group, on the other hand, showed no variation for SUN, creatinine or SUN/creatinine index when comparing pre- and post-game samples.

Taken all together, these results showed that there was a clear change in saliva composition for the game group, both in metabolite and protein concentrations, but not in the other three groups. For these reason, this study has focused attention to the set of players that were employed for the entire match, excluding the goalkeeper as explained below, and leaving out from further analysis those belonging to the other three groups.

### 2.3. Multi and Univariate Analysis of the Salivary Metabolome

**Figure 4:**
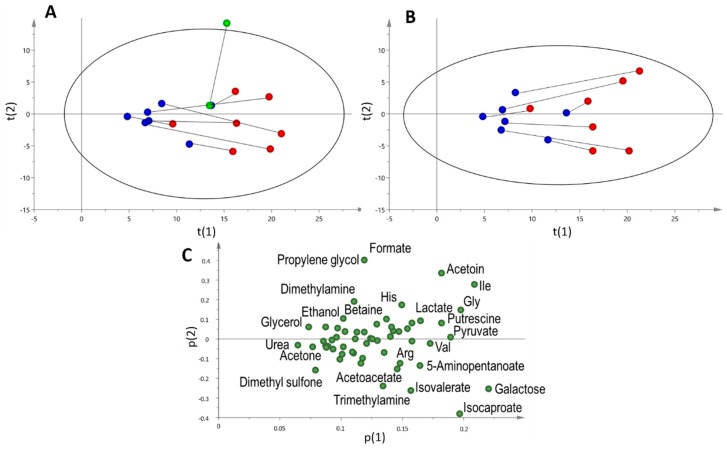
Principal component analysis (PCA) analysis of players belonging to the game group. (**A**) The score plot of pre- (blue) and post- samples (red) for field players. The lines connect the pre- and post-samples for each subject. Goalkeeper samples are depicted in green R^2^X = 0.895, Q^2^ = 0.699. (**B**) and (**C**) The score and loading plots of the game group excluding the goalkeeper. R^2^X = 0.907, Q^2^ = 0.715.

A principal component analysis (PCA) of saliva pre- and post-game samples of subjects belonging to the game group was performed. The results are shown in Figure 4A. There was a clear separation between pre- and post-samples, mainly due to an increase in the value of the first principal component. An exception to this behaviour was the goalkeeper, which showed a significant increase of the second component. From this result, it is evident that the metabolic responses of the field players and the goalkeeper are notably different, reflecting the diverse aspects of both physical and psychological roles linked to the different positions. For this reason, all the remaining analysis were performed on the game group excluding the goalkeeper (n = 7). The corresponding PCA score and loading plots for this group are shown in Figure 4B and 4C. As expected from the results of TPWS and TOMC, there was a global increase in metabolite concentrations in the post-game sample, and all metabolites showed a positive loading on p(1). However, not all metabolites showed the same increase in concentration. Compounds showing the highest weight on t(1) included galactose, isoleucine, glycine, isocaproate, pyruvate, acetoin, putrescine, valine and lactate. On the other hand, ethanolamine, methanol, O-phosphocholine, fumarate, ethanol, sucrose, dimethyl sulfone, acetone, glycerol and urea showed the smallest weight. This result showed that higher metabolite concentrations in the post-sample were not due just to a decrease in water content, but other factors regulating the concentration of the metabolites were also playing a role.

**Figure 5:**
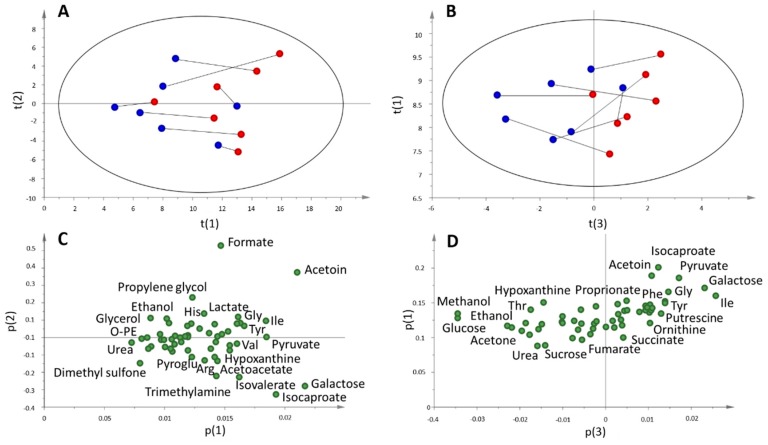
(**A**) Score plot and (**C**) loading plot for the PCA analysis of pre (blue) and post-samples (red) of the game group (n = 7) using TPWS normalization. The lines connect samples belonging to the same subject (**B**) Score plot and (**D**) loading plot for the PCA analysis of pre (blue) and post-samples (red) of the game group (n = 7) using TOMC-normalized data. Abbreviations: Arg, arginine, Gly, glycine, His, Histidine, Ile, isoleucine, O-PE, o-phosphoethanolamine, Phe, phenylalanine, Thr, threonine, Tyr, tyrosine, Val, valine.

Metabolite concentrations were then normalized using both TPWS and TOMC of each sample. In the first case, the metabolite concentration per mg of total protein was considered, and in the second, changes in the metabolite composition were monitored, keeping constant the total concentration. The results of the PCA analysis of these normalized data sets are shown in Figure 5. In the case of TPWS normalized data (Figure 5A), physical activity during the soccer match caused an increase in the first component, except for one player that showed a small variation between the two samples. In fact, although there was not a clear separation as observed with not-normalized data (Figure 4A,B), a trend along p(1) was obtained with all post-samples showing higher p(1) values than pre-samples. As with non-normalized data, all metabolites showed a positive weight on the first component, reflecting a higher response of metabolite concentrations with respect to proteins to exercise. In general, metabolites that showed the highest or lowest weight were similar to those already indicated for non-normalized data.

A different result was obtained for data normalized using TOMC (Figure 5B). The best separation between pre- and post-samples was seen in the third principal component. The corresponding loading plot showed this time a number of substances with both positive and negative contribution to t(3). Those that showed the highest negative weight were glucose, methanol, ethanol, acetone, dimethyl sulfone, taurine, glycerol, threonine, urea and isopropanol.

**Figure 6:**
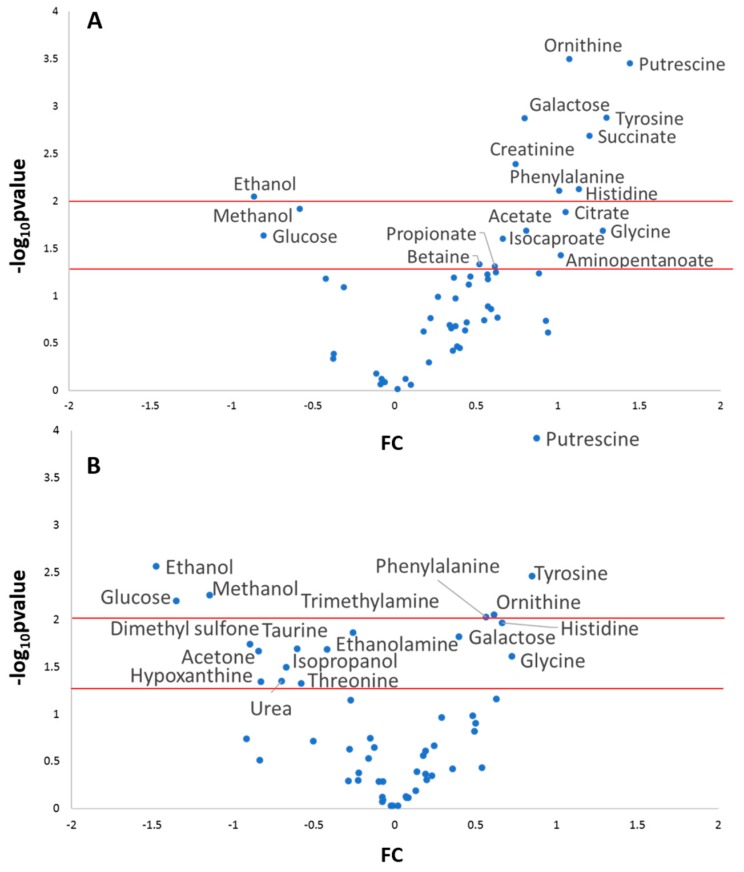
Volcano plots of changes in salivary metabolome for the game group (n = 7) using data normalized using TPWS (**A**) and TOMC (**B**). Red lines indicate the two threshold of significance (*p* = 0.01 and *p* = 0.05). The fold change, FC, was calculated as log_2_[post]/[pre].

Regarding the univariate analysis, Figure 6 shows the volcano plots for the data set of the game group normalized by TPWS and TOMC. In the first case, a number of metabolites showed a significant increment in the post-game sample, including aminoacids (ornithine, tyrosine, phenylalanine, histidine), putrescine, succinate and creatinine. A decrease was observed for ethanol, methanol and glucose. Regarding the composition of the metabolites at constant total concentration, putrescine, tyrosine, phenylalanine and ornithine showed the highest increase in the post-samples, whereas the same metabolites as before showed a decrease.

### 2.4. Salivary Lactate, Pyruvate, Succinate and Energy Related Biomarkers

**Figure 7:**
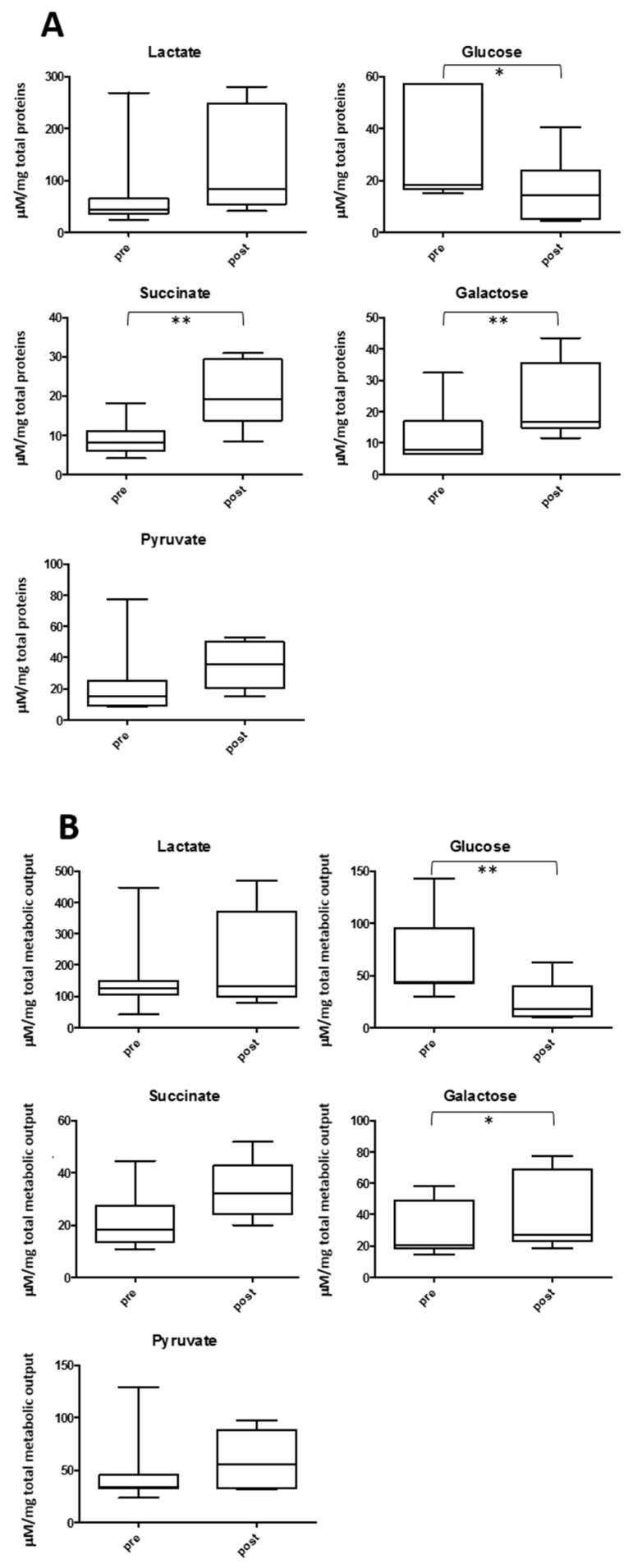
The concentration changes between pre- (**left**) and post-samples (**right**) in energy-related metabolites in saliva using TPWS (**A**) or TOMC (**B**) data normalization. In A metabolite concentrations are expressed as µM/mg of total proteins, in B as µM/total metabolic output. The boxes denote interquartile range, lines denotes median, whiskers denotes 5th and 95th percentile. * *p* < 0.05; ** *p* < 0.01.

One of the main focuses of many works on metabolomics and exercise is the change of lactate in different biofluids. Figure 7 shows the observed variation using the two normalization schemes for salivary lactate and the two closely related metabolites, succinate and pyruvate, together with glucose and galactose, other energy-related compounds. This study observed a 3.3 fold average increase of lactate (*p* = 0.02) between post- and pre-game samples when considering non-normalized saliva. However, when these concentrations were normalized using TPWS or TOMC, lactate and pyruvate did not show a significant variation, although there was an increase in succinate concentration when considering protein normalization (*p* = 0.002). Glucose and galactose levels showed the opposite variations upon exercise. There was a significant decrease in glucose (*p* value = 0.006 and 0.02 for TOMC and WSTP normalization, respectively), and an increase in galactose level in the post-samples regardless of the normalization used (*p* = 0.02 and 0.001 for TOMC and WSTP normalization, respectively).

### 2.5. Amino Acid Profile and Exercise

**Figure 8:**
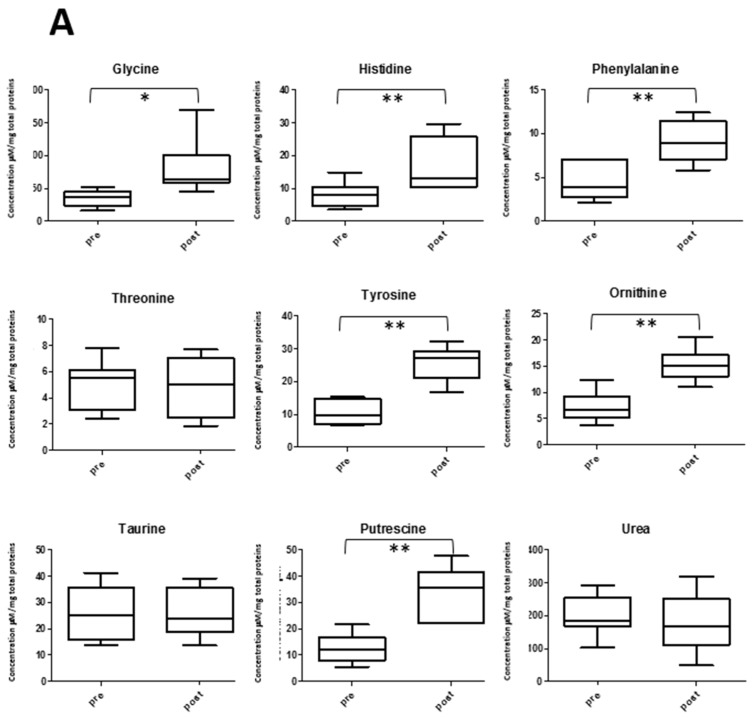

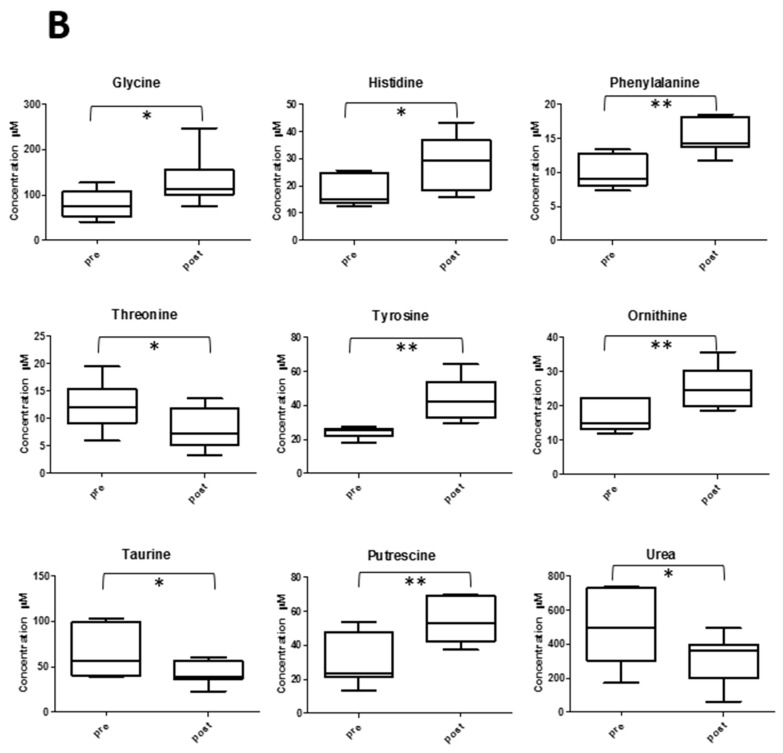
Concentration changes between pre- (**left**) and post- samples (**right**) in amino acids and related metabolites in saliva using TPWS (**A**) or TOMC (**B**) data normalization. In A metabolite concentrations are expressed as µM/mg of total proteins, in B as µM/total metabolic output. The boxes denote interquartile range, lines denotes median, whiskers denotes 5th and 95th percentile. * *p* < 0.05; ** *p* < 0.01.

Amino acids levels are important biomarkers for assessing the balance between protein synthesis and catabolism during exercise [16], and in some cases, also for specific biochemical functions linked to muscle function, development and structure [17]. Our data set contains information about 16 amino acid levels in saliva, including most of those that are the building blocks of proteins, plus ornithine and taurine. The variations between pre- and post-game were evaluated using the two normalizations schemes. These are shown in Figure 8. Glycine, histidine, phenylalanine, tyrosine and ornithine showed a significant increase both when normalizing by protein or total concentration. Threonine and taurine showed a decrease only when normalizing by TOMC. Putrescine, which is closely related to glutamate and ornithine [18], also showed a significant increase in both normalized data sets. Related to amino acid levels and protein catabolism is urea concentration variation in saliva. If samples are not normalized, urea shows a non-statistically significant increase of 1.3 fold (*p* value = 0.3). On the other hand, this study observed no changes when normalizing by TPWS and a significant decrease with respect to total metabolite concentration (*p* value = 0.04) (Figure 8).

### 2.6. Other Metabolite Concentrations Changing after the Soccer Game

**Figure 9:**
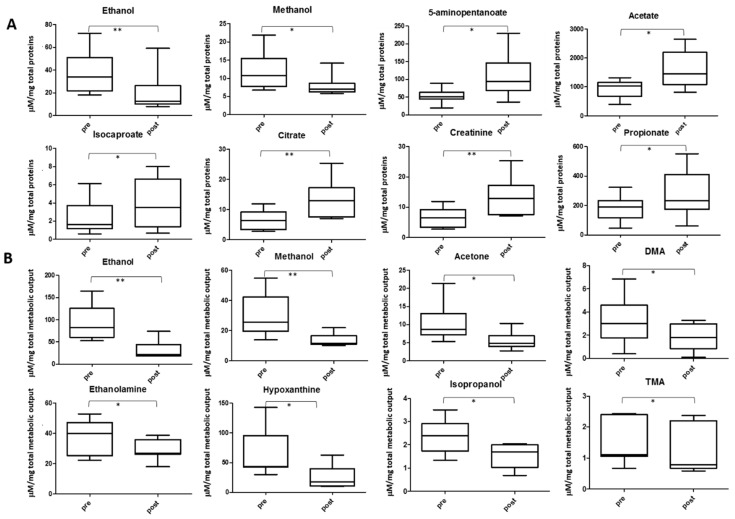
Other metabolites showing significant changes between pre- (**left**) and post-samples (**right**) when normalizing data by TPWS (**A**) or TOMC (**B**). In A metabolite concentrations are expressed as µM/mg of total proteins, in B as µM/total metabolic output. The boxes denote interquartile range, lines denotes median, whiskers denotes 5th and 95th percentile. * *p* < 0.05; ** *p* < 0.01.

Other salivary metabolites change their concentration after the football match (Figure 9). Volatile compounds like ethanol, methanol and acetone show a decreased level, partially or totally related to the higher evaporation in the mouth during exercise. With respect to total proteins, 5-aminopentanoate, acetate, isocaproate, citrate, creatinine and propionate show increased concentration. If no normalization is performed, all these increments are higher. For example, creatinine showed a 2.3 fold increment (*p* < 0.01) in non-normalized samples and 1.7 fold (*p* < 0.01) in protein-normalized saliva after the game. When considering total metabolite concentration, a significant reduction in DMA, TMA, ethanolamine, isopropanol and hypoxanthine levels in the post-game samples were observed. These reductions were not detected in the non-normalized data set.

## 3. Discussion

The use of saliva as an alternative to blood sampling for the assessment of the response to acute exercise or training was significantly stimulated by the correlation found between serum and saliva concentration of some hormones [19,20]. However, other authors have warned against a straight analysis of metabolic changes in saliva based on the known effects of physical activity on blood metabolite levels [21]. In addition, there is both a less pronounced effect on saliva metabolite concentrations compared to blood, and a shorter duration of responses [12]. This fact is particularly relevant when assessing systemic variations caused by a physical activity, not homogeneous for all players, both in intensity and timing, like a soccer match. Moreover, the time period between the last significant physical effort of a given subject and the sample uptake is variable. This can explain in part, together with the expected interindividual differences, the high variation that the authors noticed in metabolite concentrations in the post-game samples, giving rise to high standard deviation values (41 against 90 for average SD in the pre- and post-exercise metabolite concentrations for the game group, respectively). These facts suggest that more useful data for assessing relevant aspects of an athlete’s performance, training status and health can be obtained if sampling times are more frequent, for example at halftime and immediately after substitutions take place. Despite all these limitations, our results show the potential of using our measurements to assess metabolic changes correlated with physical effort. For example, a PCA analysis of saliva showed a clear separation of pre- and post-samples, mainly driven by an increase in the concentration of most metabolites. Two interesting points emerge from this analysis. One is that the goalkeeper showed a very different variation, suggesting that metabolic changes sensed by saliva were sensitive to the type of activity and the players’ role. Secondly, increments in metabolite concentrations were not homogeneous, reflecting both a decrease in water content and different stimuli that the exercise puts at play.

This study also explored two different normalization schemes to account for the higher global concentration that is expected after exercise. A significant number of works report biomarker variations without taking into account any water-content effect that is provoked either by mouth breathing or the decrease in flow rate. In other cases, results have been expressed as a ratio to total protein concentration [22]. However, this approach is only valid when no changes in the secretion rate of protein occur during or after exercise [23]. Protein concentration increases, not only because there is a reduction in water content, but also due to sympathetic activity stimulated by physical exercise [24], giving rise to an increment of certain types of proteins, not limited to amylases [11]. For this reason, in this work, two different normalizations were explored, taking into account total protein (TPWS) and total observed metabolite concentration (TOMC). In the authors’ opinion, it is worthwhile to analyse the dataset using these two normalizations, together with the non-normalized values. In the latter case, valuable information can be obtained regarding the differential concentration effect that exercise provokes on different metabolites. Whereas, a comparison of the results between the two normalization schemes can afford insights into the different impact that exercise exerts on the levels of proteins and small molecules contained in saliva.

Furthermore, values of TPWS and TOMC are useful to classify the impact of the match on the salivary metabolome and to estimate dehydration. It was observed that the group that played the entire matched showed an increase of 40% in total protein concentration, against a 20% for the group that played only the last part. These values are in very good agreement with those observed after high exercise intensity (56%) and moderate exercise intensity (14%) [11]. No increment in protein content was observed for the group that did not play at all or, interestingly, for the group that was replaced. This last result is in line with the fact that after a short period, saliva components return to their basal value.

A second way to estimate hydration status of the players is through the measurement of saliva osmolality and the flow rate [25]. Although these data were not available in the present study, a variation in TOMC reflects a change in the total number of molecules in solution, and hence its variation is correlated to osmolality changes. In this respect, it is interesting to note that increase in TOMC for the game group after the match (around 80%) was much higher than that observed for TPWS (40%). This result implies that response of metabolites is greater than the raise in protein concentration, showing that water content is not the only factor influencing the change in saliva composition after exercise.

A third indicator of hydration is the urea:creatinine ratio [14]. Values higher than 20 for the blood value was observed in the case of dehydration of hypoperfusion, mainly due to a high urea reabsorption causing a disproportionately elevated value relative to creatinine in serum. Both our measurements of this ratio in saliva, as well as other works using both NMR and MS to measure metabolite levels [15], indicated that the basal value was approximately 2-6 in saliva, significantly below that observed in blood (10–20). The concentration of both urea nitrogen and creatinine are much lower in saliva than in blood. For example, salivary creatinine concentrations were 10–15% of those observed in serum, and were found to be unrelated in healthy subjects [26]. Furthermore, exercise induced a significant decrease in the salivary ratio from 4 to 2 (*p* = 0.024). In non-normalized data, this was caused by a significant increase in creatinine after the match, a variation that was not observed when normalizing by TOMC. Other studies presented creatinine levels in saliva before and after exercise, without taking into account the change in water content, and observed either a decrease [27] or no statistically significant change [28]. Our results of an increase creatinine concentration after exercise could be related to an augmented muscle breakdown due to acute tubular necrosis caused by exercise [29].

Salivary lactate and correlated metabolites, like succinate and pyruvate, are essentially markers of the degree of tissue hypoxia reached during the exercise. Low-intensity exercises show constant lactate concentration since lactate production and removal occur with similar kinetics. On the other hand, when exercise exceeds the anaerobic threshold, a significant rise in blood lactate is normally observed [30,31]. The correlation between blood and salivary lactate is controversial. The authors observed in an earlier study no correlation between hematic lactate and salivary lactate during a small-sided game session in male soccer players [32]. Other studies reported higher correlation coefficients between lactate in blood and saliva during exercise [12,33,34,35]. One important factor that determines the degree of correlation between the two lactate levels is the time left for filtration of lactate from blood to saliva [36]. A significant increase in lactate only is found when non-normalized data are used, but not when the dataset is normalized by TPWS or TOMC. Regarding the total protein normalization, it was suggested that correlation between saliva and blood lactate was only noticed when the values were corrected by this factor [36]. This study did not observe a significant increase in lactate when normalizing by proteins. However, it is conceivable that blood lactate was incremented after the soccer match. This lack of correlation can be in part explained by the different type of exercise, and more importantly, the variable time between the last high intense effort and the post-game sample uptake. Based on all these considerations, the change in salivary lactate obtained in these conditions may be only of limited value if it is intended to define training routines in order to enhance sport performance.

Two other energy related metabolites show an interesting opposite variation upon exercise. They are glucose and galactose. While the first showed a significant decrease, galactose levels were higher in the post-game samples, using both types of data normalization. It is tempting to speculate that this result is correlated to known differences between the two carbohydrates and exercise demand. For example, it was shown that the oxidation rate of orally ingested galactose was maximally 50% of the oxidation rate of a comparable amount of orally ingested glucose during 120 min of exercise [37]. A different role of galactose and glucose in liver glycogen replenishment was also described, using drinks consumed during short-term post-exercise recovery [38].

Metabolites like amino acids, urea and putrescine reflect physical effort on the body metabolism. Whereas the total venous plasma amino acid concentration after a 70 km cross-country ski race was observed to fall to approximately 60% of the pre-race level [16], salivary amino acid concentrations were observed to slightly increase after physical exercise [39]. It was observed that most amino acid levels were not significantly changed after the soccer match, with a few showing an increase, that is, glycine, histidine, phenylalanine, threonine and tyrosine. The elevation in aromatic amino acid levels is in line with that observed in blood [16], suggesting an interesting correlation between blood and saliva for these metabolites. Other amino acids changing after exercise is taurine, for which a decrease in post-game samples was observed. Several studies have focused on the effects of taurine during physical activity [17]. A diminished level of taurine was observed in rat skeletal muscles after exercise [40], and in blood in a group of mice that behaved as spontaneous wheel runners [41]. A similar decrease in salivary taurine levels was observed in young soccer players after a training session, and also correlated with distances covered by players during a game [32]. The authors also observed an increase of putrescine in saliva, probably related to the known effect of polyamine accumulation in the skeletal muscle after physical exercise [42]. It is thought that polyamine expression is important in aiding slow muscle fibers recovering from exhaustive exercise.

Hypoxanthine, a degradation product of ATP, was reduced in saliva after the soccer match. Purine concentrations were observed to be reduced in muscle and plasma, together with a decreased urinary excretion, after sprint training [43]. This reduction in hypoxanthine levels in blood, muscle, urine and now saliva, likely represents a training-induced adaptation to minimize the loss of purines from skeletal muscle. This adaptation is advantageous in reducing the extent of replacement of the muscle nucleotide pool via the metabolically expensive purine de novo synthesis pathway.

Metabolomics studies, as the one here presented, offer the possibility of measuring the levels of a high number of metabolites in biofluids. In particular, when saliva is used to monitor the extent of metabolic response to physical exercise, having access to a panel of different biomarkers instead of just a small number, is of vital importance. When evaluating the systemic response to this type of stimulus, not only the competition itself determines the final effect, but also diet, hydration, training and global health of the athlete. The assessment of biomarkers should include select, diverse and well-validated markers of performance, health and recovery, which in turn can offer information regarding muscle status and oxygen transport, nutritional and hydration status, inflammation injury risk and muscle damage. From a practical point of view, however, it is necessary to obtain additional measurements sensitive to exercise intensity, such as the total distance covered, and when appropriate even respirometry, in order to normalize the observed metabolic changes with respect to the actual physical effort. On the other hand, the short time of the effect on saliva precludes analysis of the partial game group, and probably renders heterogeneous the measured changes provoked by the game. If used as biofluid, with all the recognized advantages, saliva needs to be taken shortly after the game, and in the case of players that are substituted, immediately after they finish their physical activity. Only a global vision of many biomarkers can help in understanding the complex reasons that govern metabolic response to exercise.

## 4. Materials and Methods

### 4.1. Study Participants

Seventeen Res Roma professional female football players participated in the study. The sampling was performed during a *Coppa Italia* soccer match (Res Roma-Verona). The game had an overall duration of 90 min. Saliva samples were collected before and after the game. The study was approved by the Ethics Committee of the University Hospital Tor Vergata (ID number 41.17) and all subjects signed an informed consent. The study was conducted in accordance with the Declaration of Helsinki.

### 4.2. Saliva Sampling

Unstimulated whole saliva was collected for passive drooling before and after soccer game. The participants were seated with their head tilted forward, allowing saliva to pool in front of the mouth. Saliva was allowed to be dribbled out of the mouth into polypropylene tubes until a sufficient amount (i.e., 2 mL) was obtained. The saliva samples were centrifuged at 4000× *g* at 4 °C for 30 min, aliquoted and stored at −80 °C until use.

### 4.3. Total Protein Quantification

Saliva total protein concentration was measured using a standard Bradford assay (Bio-Rad Protein Assay kit II, Bio-Rad, München, Germany) [44]. Absorbance readings were run with Infinite^®^ M200 microplate reader by TECAN (Grödig, Austria).

### 4.4. Saliva Protocol Preparation by NMR Spectroscopy

Saliva samples were prepared using a protocol previously reported by Cicero and colleagues [32]. Briefly, saliva samples were deproteinized by ultrafiltration with 0.5 mL Amicon Ultra 3kDa cut-off centrifugal filter units. Prior to filtration, filter devices were washed four times with 500 µL of water and centrifuged at 13,800× *g* for 25 min at 4 °C in order to remove residual glycerol bound to the filter membrane. The washing procedure was repeated until the control by NMR spectroscopy of the filtrate showed no residual presence of glycerol. A volume of 500 µL of the saliva sample was transferred into the filter device and centrifuged at 13,800× *g* at 4 °C for 90 min. An amount of 400 µL of filtrate was diluted with 100 µL of buffer (250 mM of phosphate buffer pH 7.4 containing 1mM 3-(trimethylsilyl)propionic acid-d_4_ sodium salt, TSP, 2% NaN_3_, 10% D_2_O). The final solution was mixed and transferred to a 5 mm NMR tube.

### 4.5. NMR Spectroscopy Acquisition

Spectra were acquired on Bruker 600 MHz Avance spectrometer equipped with an inverse 5mm BBI probe with z-gradients. ^1^H-NMR spectra were acquired at 25 °C using the NOESYPR1D (1D Nuclear Overhauser effect spectroscopy with water pre-saturation) pulse sequence (RD-90°-*t*_1_-90°-*t*_m_-90°-acquire) with *t*_1_ = 4 µs, *t*_m_ = 100 ms, spectral width of 12 ppm, acquisition time of 2 s, relaxation delay of 3 s, 2048 transients, 4 dummy scans. All spectra were acquired at 298 K. Water saturation was performed using a continuous-wave field, both during relaxation delay and mixing time. All FIDs were zero-filled to 128k data point and subjected to line broadening of 0.5 Hz. All spectra were phased and baseline-corrected manually before being used for the estimation of metabolite concentrations.

### 4.6. Identification and Quantification of Metabolite

NMR spectra acquired were imported in Chenomx NMR suite software (version 8.1). The software allowed the identification and quantification of metabolites present in Chenomx library. As an internal standard, TSP with a final concentration of 0.2 mM was used. All spectra were calibrated, phased and the baseline-corrected.

### 4.7. Multivariate Data Analysis

Multivariate data analysis was carried out using SIMCA-P (version 14 Umetrics AB, Umea, Sweden). The data were median scaled, dividing each variable by the median of that metabolite in all samples prior to analysis. Principal component analysis (PCA) was performed on the resulting data matrix. PCA was run to obtain a general overview of the variance of the NMR sample. This method provides the possibility to detect and exclude outliers, defined as observation outside the 95% confidence region of the model. The robustness of the models is indicated by the following parameters: R^2^X, variation of X explained by the model and Q^2^, goodness of prediction. R^2^ varies between 0 and 1, Q^2^ varies between −1 and 1.

### 4.8. Univariate Data Analysis

Metabolites concentration values were also analysed by univariate analysis using both not-normalized and normalized data. Two normalization methods were used: Probabilistic quotient normalization method [45] and total protein normalization. The first normalization method is based on the calculation of a most probable dilution factor by looking at the distribution of the quotients of the amplitudes of a test spectrum by those of a reference spectrum. The reference spectrum can be a single spectrum of the study, a golden reference spectrum from a database, or a calculated median or mean spectrum on the basis of all spectra of the study or on the basis of a subset of the study. [45] In this work, as reference spectrum, a calculated median was chosen. Briefly, for each metabolite, concentrations median in all samples were calculated and each concentration value was divided by the median. The normalization factor was then obtained calculating the median of this quotient for each sample. For protein normalization, each concentration value was divided by the total protein content calculated for each sample with Bradford assay (see above). The normalization factors, in both normalization methods, were calculated for each sample and not for individuals. For volcano plots analysis, for each metabolite, a fold change value, FC, was calculated as the binary logarithm of the ratio between average post- and pre- samples concentrations as expressed in the following equation:

FC = log21N∑i([Post]i[A]i)1N∑i([Pre]i[A′]i)

where A and A’ are mg of total proteins for TPWS normalization or probabilistic quotients for TOMC normalization respectively for post- and pre samples, N = number of samples, [Post]_i_ = i-th metabolite concentration in post-samples, [Pre]_i_ = i-th metabolite concentration in pre samples.

## 5. Conclusions

In this work, the authors explored the limits and advantages of using saliva to observe the metabolic response to physical exercise developed during an official soccer match. A relevant question is the data normalization criterion, because saliva is a biofluid that changes significantly, not only its composition, but also the water content after exercise. Most of the literature data about metabolite level changes are informed without any normalization, and hence reflecting partly or significantly, a change in water content rather than a systemic metabolic variation. This study has shown that both proteins and total metabolite concentrations were useful for normalizing the data, and different conclusions can be drawn using both as reference. Despite the heterogeneous impact of exercise that each player performs during a soccer match, and the diverse time that passes from the last sprint and the moment the sample is taken, several salivary biomarkers show responses to exercise. However, the short time of the effect on saliva precluded the analysis of the partial game group, and probably rendered heterogeneous the measured changes provoked by the game. If used as biofluid, with all the recognized advantages, saliva needs to be taken shortly after the game, and in the case of players that are substituted, immediately after they finish their physical activity. Other studies may add to this point and expand the use of saliva as a source of information in the field of metabolomics and physical exercise.

## Figures and Tables

**Table 1:** Main characteristics of the female soccer players involved in the study.

Outlines	Game Group	Enter Group	Exit Group	No Game Group
Players	8/17	3/17	3/17	3/17
Age (years)	23 ± 5	18 ± 1	23 ± 8	23 ± 6
Weight (kg)	61 ± 9	55 ± 1	59 ± 5	67 ± 6
BMI (kg/m^2^)	24 ± 2	22 ± 2	23 ± 3	24 ± 3

**Table 2:** The average concentrations (µM) with relative standard deviations of saliva metabolites in pre-game samples as determined by ^1^H NMR.

Metabolite ID	Pre-Samples	Metabolite ID	Pre-Samples
5-Aminopentanoate	99 ± 58	Isocaproate	4 ± 3
Acetate	1784 ± 929	Isoleucine	3 ± 2
Acetoacetate	3 ± 3	Isopropanol	2 ± 1
Acetoin	10 ± 7	Isovalerate	6 ± 4
Acetone	9 ± 3	Lactate	158 ± 152
Alanine	25 ± 19	Leucine	7 ± 4
Arginine	13 ± 10	Methanol	29 ± 29
Aspartate	18 ± 9	O-Phosphocholine	4 ± 1
Betaine	2 ± 1	O-Phosphoethanolamine	39 ± 21
Butyrate	42 ± 27	Ornithine	15 ± 8
Choline	5 ± 3	Phenylalanine	9 ± 5
Citrate	18 ± 9	Proline	39 ± 29
Creatine	10 ± 5	Propionate	360 ± 229
Creatinine	3 ± 1	Propylene glycol	12 ± 9
Dimethylamine	2 ± 1	Putrescine	25 ± 16
Dimethyl sulfone	3 ± 2	Pyroglutamate	8 ± 10
Ethanol	135 ± 242	Pyruvate	37 ± 42
Ethanolamine	34 ± 20	Sarcosine	2 ± 1
Formate	188 ± 318	Serine	32 ± 18
Fumarate	0.4 ± 0.2	Succinate	23 ± 12
Galactose	23 ± 24	Sucrose	11 ± 13
Glucose	60 ± 45	Taurine	53 ± 24
Glutamate	58 ± 37	Threonine	9 ± 6
Glutamine	18 ± 14	Trimethylamine	1.3 ± 0.9
Glycerol	267 ± 145	Tyrosine	20 ± 9
Glycine	68 ± 43	Urea	561 ± 226
Histidine	16 ± 9	Urocanate	3 ± 2
Hypoxanthine	4 ± 3	Valine	6 ± 4

**Table 3:** Reference values for urea nitrogen (UN), creatinine and the ratio UN/creatinine in serum and saliva.

Dehydration Index	Serum	Saliva	
	Literature ^a^	Literature ^b^	This Work ^c^
UN [mg/dL]	7-20	0.22 ± 0.18	0.20 ± 0.08
Creatinine [mg/dL]	0.7-1.2	0.06 ± 0.04	0.04 ± 0.01
UN/creatinine	10-20	4 ± 2	6 ± 2

a. Reference [14]. b. Reference [15]. c. Average values and standard deviations calculated for all 17 players (pre-game samples).
